# Case Report: A rare presentation and diagnosis of gingival melanoacanthoma caused by teeth whitening strips: A Case Report

**DOI:** 10.12688/f1000research.27999.2

**Published:** 2020-12-21

**Authors:** Hamad Albagieh, Ashwag Aloyouny, Shatha Alharthi

**Affiliations:** 1 Oral Medicine and Diagnostic Science Department, College of Dentistry, King Saud University, Riyadh, Saudi Arabia; 2 Basic Dental Science Department, College of Dentistry, Princess Nourah bint Abdulrahman University, Riyadh, Saudi Arabia; 3 Preventive Dental Science Department, College of Dentistry, Princess Nourah bint Abdulrahman University, Riyadh, Saudi Arabia

**Keywords:** Oral Melanoacanthoma, gingival hyperpigmentation, oral pigmented lesion, teeth whitening strips.

## Abstract

**Background:** Oral melanoacanthoma is not common. It occurs mostly on the buccal mucosa. Since it happens suddenly and progresses rapidly, it clinically resembles melanoma. Melanoacanthoma occurs in regions susceptible to trauma. The clinical presentation of the lesion is not enough to diagnose it; therefore, tissue biopsy is necessary to exclude malignancy.

**Case report:** We report a case of oral melanoacanthoma in a rare mucosal location in a 21-year old female patient in whom generalized gingival melanoacanthoma was related to the use of the teeth whitening strips. This irritating factor increased melanocyte activity in the gingival tissues and labial mucosa.

**Discussion:** Oral melanoacanthoma is a rarely encountered pigmented lesion in the oral cavity and is especially uncommon in the gingiva. It is a reactive lesion affecting the mucous membranes with no risk of malignant transformation. This case report shows that teeth whitening strips may trigger oral melanoacanthoma in susceptible individuals. Long-term irritation of the oral tissues may increase the number of dendritic melanocytes throughout the epithelium and accordingly increase the brown pigmentation of the oral cavity.  Eliminating all possible local sources of irritation and ruling out other causative factors are the standard first step in the treatment of oral melanoacanthoma.

**Conclusions:** This case shows the importance of including oral melanoacanthoma in the differential diagnosis of diffuse gingival pigmented lesions.

## Introduction

Oral Melanoacanthoma is a rare, pigmented lesion that usually occurs on the buccal mucosa. The most common site of oral melanoacanthoma is the buccal mucosa (51.4%), followed by the palate (22.2%), and lips (15.2%). The gingiva is least affected by melanoacanthoma (5.6%) ^1^. A review of the literature identified only a few cases with generalized, diffuse, multiple, upper and lower gingival melanoacanthoma ^2^. Clinical differentiation between benign and malignant oral pigmented lesions is very difficult at early stages. Generalized gingival melanoacanthoma is extremely rare, therefore, tissue biopsy is highly recommended to differentiate between melanoacanthoma and melanoma. We present a rare case of melanoacanthoma in the gingivae and the labial mucosae triggered by teeth whitening strips in a 21-year-old female patient. The work is reported in line with the CARE criteria ^3^.

## Case report

**Figure 1.**
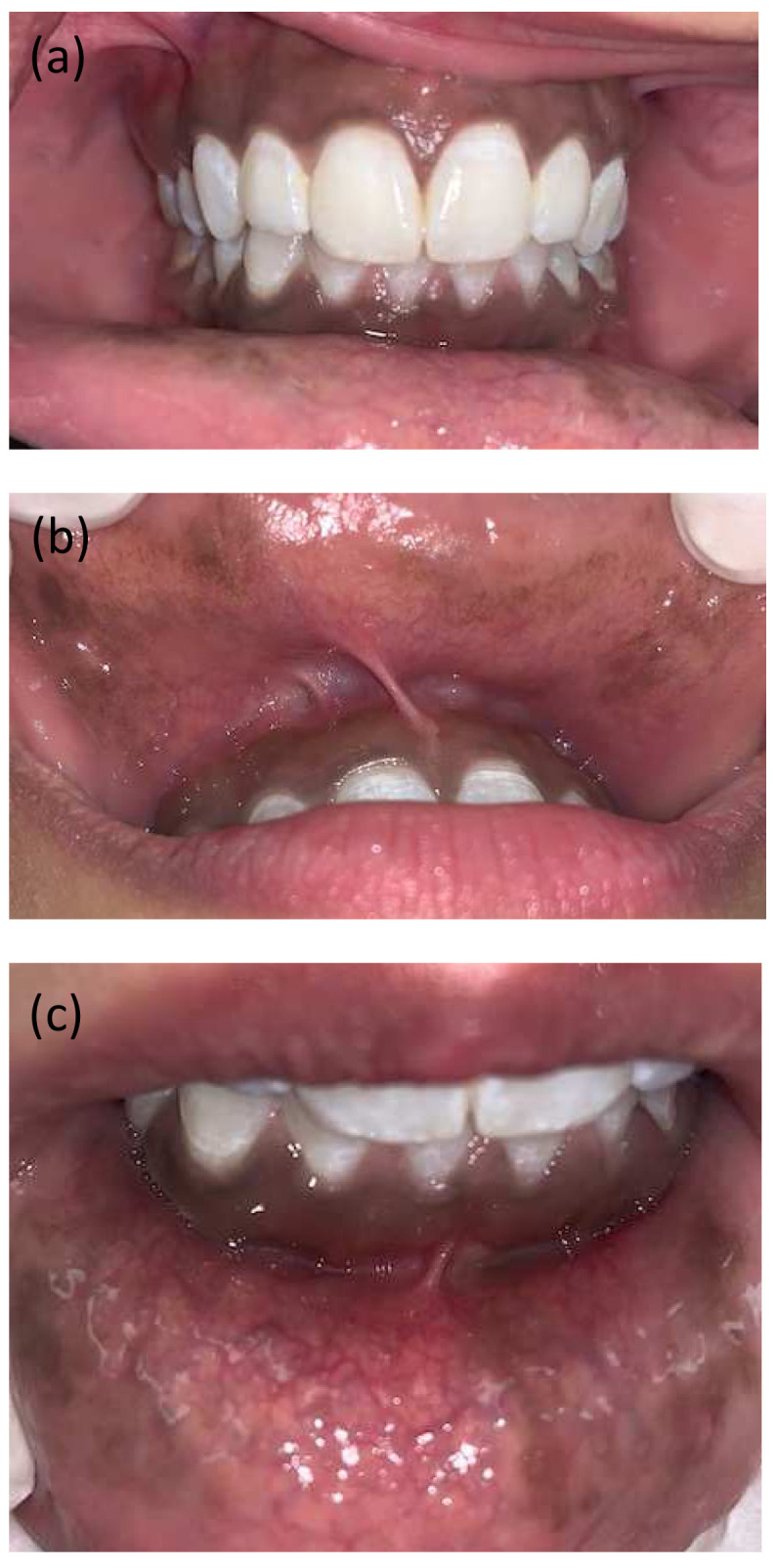
Clinical photographs of oral melanoacanthoma. Macular brown pigmentation with irregular margins involving ( **a**) upper and lower attached and free gingivae; ( **b**) upper labial mucosa; ( **c**) lower labial mucosa.

The patient of this case report was a 21-year-old female, Saudi Arabian college student. She was referred by her dentist in July 2018 to an oral medicine specialist for evaluation of a three-month-history of remarkable intraoral, diffuse hyperpigmentation of the upper and lower gingivae and labial mucosae. She reported that the hyperpigmentation appeared suddenly and had rapidly increased in size. The patient reported using teeth whitening strips for six weeks before the intraoral pigmentation happened. Her past medical history revealed hypothyroidism for three years for which she had been taking 100 mcg/day of levothyroxine sodium. She was not on any other mediation. She was not aware of any relevant family history of extensive brown oral pigmentation. She denied bronzing of her skin. She reported no history of mental health disorders and denied the use of tobacco products. The patient was referred to an endocrinologist to rule out systemic diseases such as Addison’s disease. Accordingly, a blood test was carried out and the results were all within normal limits; red blood cell count of 4.99 × 10 ^6^/μL, platelet count of 259 × 10 ^3^/μL, hemoglobin count of 13.3 g/dL, white blood cell count of 8.88 × 10 ^3^/μL, lymphocytes 33.6%, segmented neutrophil 54.6%, ferritin 31.11 ng/mL, iron 14.6 umol/L, FT4 17.67 ng/dL, TSH 0.079 mIU/L, Vitamin D 65 ng/mL serum Na 139 mmol/L, serum K 4.2 mmol/L, ACTH 26 pg/ml and cortisol 184.9 nmol/L. A physical examination revealed no skin pigmentation and an extraoral examination showed no significant findings. An intraoral examination showed asymptomatic, diffuse, smooth, macular blackish-brown pigmentation with irregular margins on both the upper and lower attached and free gingivae, and upper and lower labial mucosae ( Figures 1a–1c).

**Figure 2.**
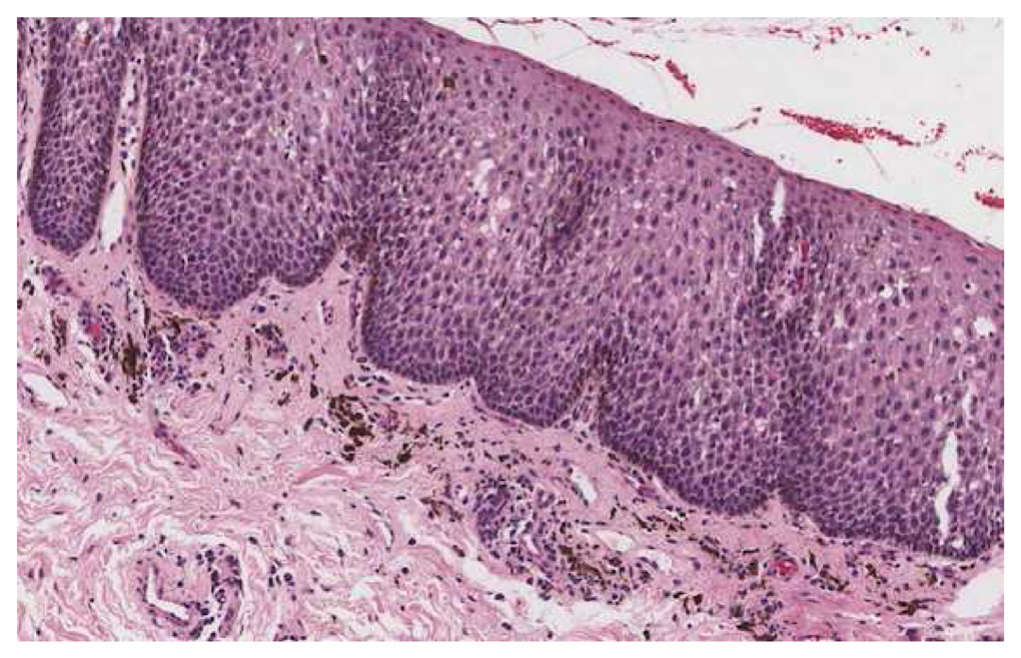
Microphotograph of the stained biopsy tissue. Microphotograph of hematoxylin and eosins stained tissue shows the parakeratinized, hyperplastic, stratified squamous epithelium with acanthosis, and long rete ridges. Many benign dendritic melanocytes with dendritic processes can be seen distributed in the epithelium, as well as melanin deposits in the lamina propria.

**Figure 3.**
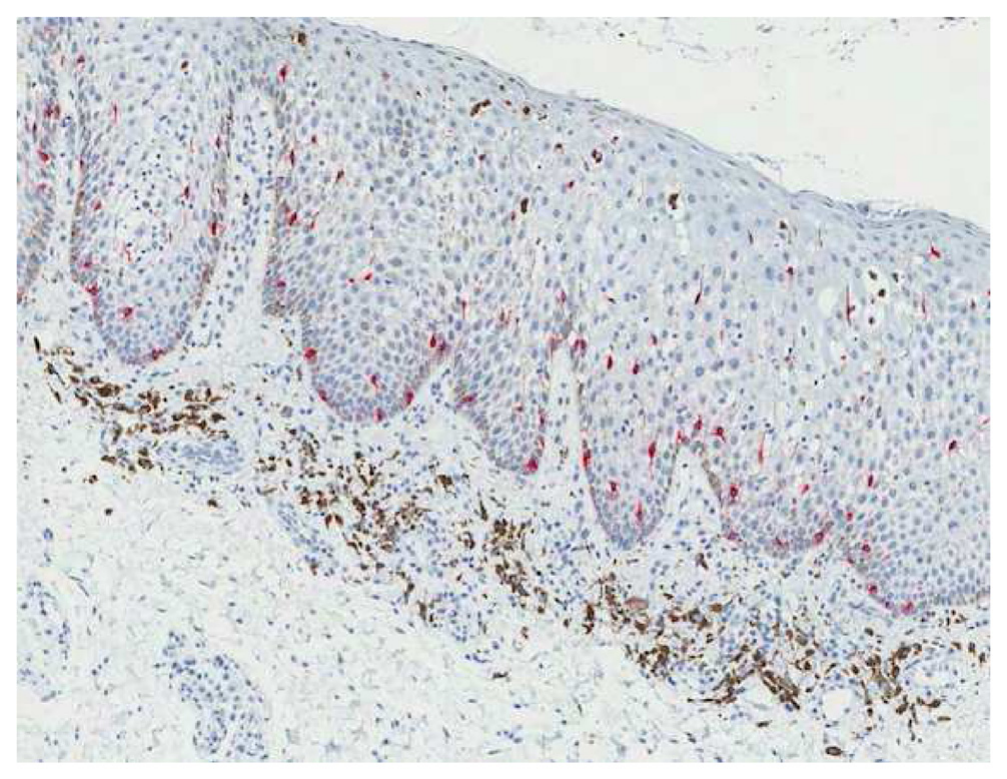
Microphotograph of Melan-A stained biopsy tissue. Microphotograph of Melan-A stained biopsy tissue shows melanotic hyperplasia in the epithelium.

The clinical presentation and the widespread nature of the lesion were worrisome to the clinician; therefore an incisional biopsy was performed. The goals and expectations of the procedure were discussed with the patient as well as the potential risks for surgical and post-surgical complications such as bleeding, swelling, discomfort, infection and scarring. Written informed consent was obtained from the patient. After applying 1.8 mL of the anesthetic solution (lidocaine HCL 2% and epinephrine 1:100,000) via intraoral injection to the gingival area of tooth No. 33, a 3 mm section of gingival tissue was removed at the apical area of tooth No. 33, at the darkest pigmented spot of the lesion and a bit away from the esthetic zone. The incised tissue was blackish-brown in colour and measured 0.3 × 0.2 × 0.1 cm. The gross specimen was fixed in 10% neutral buffered formalin and then submitted as one piece in one cassette for histopathology examination. The microscopic analysis of the hematoxylin and eosin (H&E) stained sections showed a hyperorthokeratinized, hyperplastic, stratified squamous epithelium revealing acanthosis, and long rete ridges. Many benign dendritic melanocytes with pigment-laden dendritic processes were distributed in the epithelium. In addition, melanin pigmentation of basal cell layer was noted with evidence of melanin deposits in the lamina propria ( Figure 2). A microphotograph of Melan-A stained tissue shows melanotic hyperplasia in the epithelium ( Figure 3).

**Figure 4.**
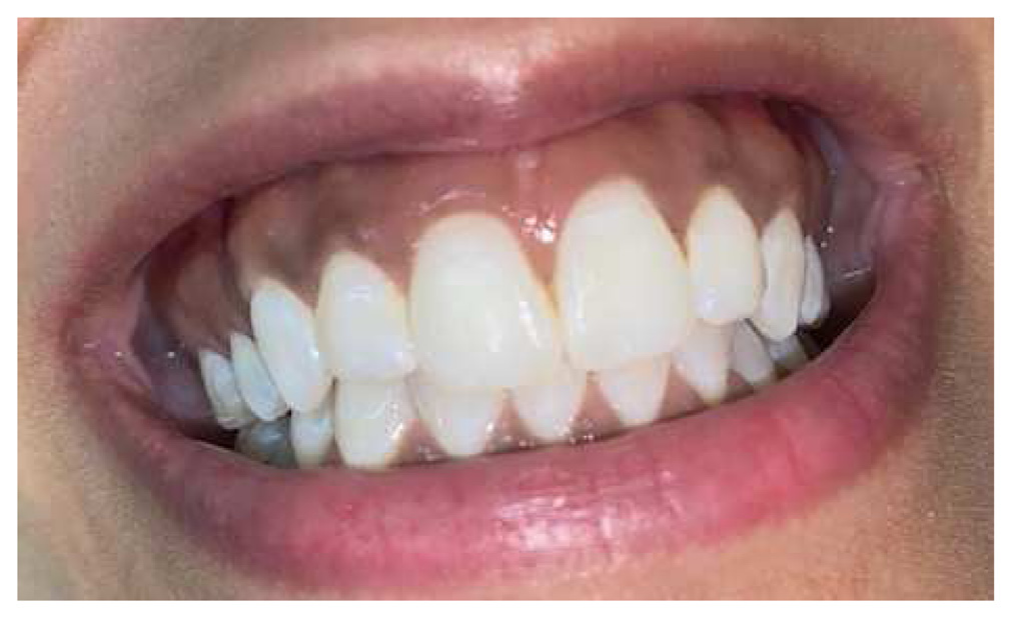
Follow-up clinical photograph. An eight-month-follow up visit revealed that the brown pigmentation had faded gradually.

In light of the patient history, clinical presentation, and the histopathology report of the incisional biopsy, the final diagnosis of oral melanoacanthoma was confirmed. The patient was reassured of the benign nature of the lesion and she was advised to stop using the teeth whitening strips. At 8-months follow up, the lesion had gradually faded ( Figure 4).

## Discussion

Cutaneous melanoacanthoma was first reported in 1927, yet oral melanoacanthoma was not described until 1978 ^4^. Melanoacanthoma was named by Mishima and Pinkus in 1960 ^5, 6^. It shares some of the alarming characteristic features of malignant melanoma such as sudden appearance and rapid growth rate. It is described as asymptomatic, solitary or multifocal, and diffuse, with ill-defined areas of dark brown to black pigmentation, a flat macule or slightly raised, and is usually greater than 1 cm in diameter. It usually occurs in teenage to middle-aged women with dark skin pigmentation ^7^.

The exact pathophysiology of oral melanoacanthoma is undetermined; however, the clinical manifestations of the lesion is suggestive of a reactive origin, since it occurs mainly in regions liable to trauma ^8^. The oral melanoacanthoma, in this reported case, occurred on the soft tissue areas that came in close contact with the teeth whitening strips. After excluding all other potential causes, the author believed that the teeth whitening strips could have participated in the oral pigmented lesion in this patient, resulting in diffuse hyperpigmentation. These teeth whitening strips could have been a source of irritation to the tissues as they contain a gel that has active chemical ingredients such as hydrogen peroxide. Prolonged exposure to high concentrations of hydrogen peroxide can damage oral soft tissues ^9^.

The differential diagnoses of oral melanoacanthoma includes: melanoma, Addison’s disease, McCune-Albright syndrome, Peutz-Jeghers syndrome, physiologic racial pigmentation, post-inflammatory lichen planus, acquired melanocytic nevus, and oral melanotic macules ^10^. Gingival pigmentation may also be caused by amalgam material, chewing tobacco (khat), gingival tattoo, graphite implantation, and several hygiene products. Moreover, utilizing different heavy metals and some medically prescribed drugs could participate in the presence of pigmented lesions in the mouth ^2^. Fortunately, oral melanoacanthoma has an excellent prognosis; in most of the reported cases, the pigmented lesions start to fade gradually either after removing the causative factor such as dental hygiene products or tobacco products, or following minor trauma such as tissue biopsy or sharp food injury. In the present case, pigmentation gradually disappeared after discontinuation of the whitening strips. Eliminating all possible local sources of irritation and ruling out other causative factors are the standard first step in the oral melanoacanthoma therapy.

This case study has many strengths; the clinical examination was done thoroughly, the laboratory result was reviewed carefully, and complete data was collected. After that, a tissue biopsy from the lesion was performed to determine the condition and based on the histopathology report, the causative factor was eliminated. As a result, the lesion regressed gradually.

## Conclusion

Oral melanoacanthomas are rarely encountered pigmented lesions in the oral cavity. They are especially rare in the gingival region. These pigmented lesions should be biopsied and carefully analyzed under the microscope to rule out a diagnosis of a malignant lesion such as melanoma. This case report highlights the important role of dentists to include oral melanoacanthoma in the differential diagnosis of diffuse gingival pigmented lesions.

## Patient perspective

The patients reported that the disappearance of the brown discoloration had a good impact on the esthetic appearance of her smile which allowed her to regain confidence.

## Consent

Written informed consent was obtained from the patient for publication of this case report and accompanying images.

## Data availability statement

All data underlying the results are available as part of the article and no additional source data are required.
