# Supplementary material for: Case Report: A rare presentation and diagnosis of gingival melanoacanthoma caused by teeth whitening strips: A Case Report
Source: F1000Res. 2020 Dec 21;9:1452. Originally published 2020 Dec 14. [Version 2] doi: 10.12688/f1000research.27999.2 (PMC7780343; doi:10.12688/f1000research.27999.2)
Supplement: Peer review history

# Peer review: Reviewer response for version 1

**DOI:** 10.5256/f1000research.30968.r76102

**Published:** 2020-12-21

**Authors:** Lujain Homeida

**Affiliations:** 1 Faculty of Dentistry, Umm Al-Qura University, Makkah, Saudi Arabia

I reviewed the manuscript entitled: " Case Report: A rare presentation and diagnosis of gingival melanoacanthoma caused by teeth whitening strips" and found it of interest to the readers of F1000Research.

 In this paper, they reported a case of a rare pigmented mucosal melanoacanthoma with uncommon trigger. The importance of this case arises from its resemblance clinically to malignant melanoma such as sudden appearance and rapid growth rate. However, melanoacnthoma has no risk of malignant transformation. They concluded by emphasizing on considering oral melanoacanthoma in the differential diagnosis of diffuse gingival pigmented lesion.

 This case report highlights the importance of investigating cases with oral pigmented lesions through detailed history taking and clinical work up to rule out any potential malignancies. The presented clinical evaluation and management of this case is excellent. I found this paper very relevant to the scope of this journal. The only point I would arise here is that they referred to some relatively old references and I would kindly advise them to use more updated references.

Are enough details provided of any physical examination and diagnostic tests, treatment given and outcomes?

Yes

Is the case presented with sufficient detail to be useful for other practitioners?

Yes

Is sufficient discussion included of the importance of the findings and their relevance to future understanding of disease processes, diagnosis or treatment?

Yes

Is the background of the case’s history and progression described in sufficient detail?

Yes

Reviewer Expertise:

Oral medicine/ TMDs/Medcially compromised patients.

I confirm that I have read this submission and believe that I have an appropriate level of expertise to confirm that it is of an acceptable scientific standard.

# Peer review: Reviewer response for version 1

**DOI:** 10.5256/f1000research.30968.r76096

**Published:** 2020-12-15

**Authors:** Mahnaz Fatahzadeh

**Affiliations:** 1 Department of Diagnostic Sciences, Rutgers School of Dental Medicine, Newark, USA

The case is well-documented and the article is well-written. Perhaps, a reviewer in that field may offer additional insights regarding the photomicrographs.

 I have made a few minor suggestions to improve the article as follows:

- Title: It may be more appropriate to change it to "A rare presentation of gingival melanoacanthoma caused by teeth whitening strips: A Case Report".
- Abstract, case report paragraph: Please combine 1st and 2nd sentence as follows" ..... female patient in whom generalized gingival melanaoacanthoma was related to the use of teeth whitening strips."
- Abstract, discussion paragraph, last sentence: change to "..... first step in treatment of oral melanoacanthoma."
- Page 3, case report, 1st paragraph: It is appropriate to include if patient reported bronzing of her skin or not.
- Page 3, case report, 1st paragraph: If available, please include the result of typical tests for Addison's disease (serum Na, K, cortisol, ACTH and their normal ranges).
- Page 3, case report, second paragraph: I agree that biopsy was warranted to determine the nature of diffuse oral pigmentation in this case. However; melanoma would be a less likely concern in this case. Oral melanoma is rare and presentation of gingival melanoma in both jaws is even more rare.
- Page 4, discussion, second paragraph: correct the spelling for the word "diffuse".
- Page 4, discussion, third paragraph: Did the mandibular gingival pigmentation also resolve over time? If so, it is more likely to be due to discontinuation of whitening strips rather than biopsy performed in maxilla. 

Are enough details provided of any physical examination and diagnostic tests, treatment given and outcomes?

Yes

Is the case presented with sufficient detail to be useful for other practitioners?

Yes

Is sufficient discussion included of the importance of the findings and their relevance to future understanding of disease processes, diagnosis or treatment?

Yes

Is the background of the case’s history and progression described in sufficient detail?

Yes

Reviewer Expertise:

Oral medicine

I confirm that I have read this submission and believe that I have an appropriate level of expertise to confirm that it is of an acceptable scientific standard.
